# Development and Evaluation of Algorithms for Breath Alcohol Screening

**DOI:** 10.3390/s16040469

**Published:** 2016-04-01

**Authors:** Jonas Ljungblad, Bertil Hök, Mikael Ekström

**Affiliations:** 1 Hök Instrument AB, Flottiljgatan 49, SE-721 31 Västerås, Sweden; bertil.hok@hokinstrument.com; 2 School of Innovation, Design and Engineering, Mälardalen University, Box 883, SE-721 23 Västerås, Sweden; mikael.ekstrom@mdh.se

**Keywords:** breath alcohol screening, contactless measurement, tracer gas measurement

## Abstract

Breath alcohol screening is important for traffic safety, access control and other areas of health promotion. A family of sensor devices useful for these purposes is being developed and evaluated. This paper is focusing on algorithms for the determination of breath alcohol concentration in diluted breath samples using carbon dioxide to compensate for the dilution. The examined algorithms make use of signal averaging, weighting and personalization to reduce estimation errors. Evaluation has been performed by using data from a previously conducted human study. It is concluded that these features in combination will significantly reduce the random error compared to the signal averaging algorithm taken alone.

## 1. Introduction

Breath alcohol screening is of significant importance as a means of preventing intoxicated drivers from causing serious traffic accidents. It is also highly relevant for access control to restricted areas, such as nuclear power plants, process industry, and mining premises. Alcohol screening may also be used in other situations for the purpose of health promotion.

Devices for the determination of breath alcohol concentration (BrAC) are commercially available for screening and evidential purposes, and alcohol interlocks are being increasingly used [1]. However, there is a need for radical improvement in order to make such devices acceptable on a larger scale, in line with the DADSS initiative (driver alcohol detection system for safety) [2,3]. The DADSS initiative is a research partnership between NHTSA (National Highway Traffic Safety Administration) and ACTS (Automotive Coalition for Traffic Safety) aimed to prevent alcohol impaired driving by technological advancement towards unobtrusive technology. Deployment of unobtrusive BrAC screening on a large scale could potentially save tens of thousands of lives every year by preventing drunk driving.

Our research towards less obtrusive sensor systems for breath alcohol screening started in 2005. The envisioned system will unobtrusively and accurately detect alcohol in the driver’s breath before the vehicle may be started, or while driving. In earlier publications, we have demonstrated methods and system solutions for contactless determination of BrAC [4,5] in screening applications where sobriety is expected to be the norm. The physiological rationale of using a tracer gas, e.g., carbon dioxide (CO${}_{2}$), for contactless determination was examined [6], and the usefulness of this technique in patients with reduced consciousness was demonstrated [7]. Recently, further progress towards unobtrusive and highly accurate BrAC determination in automotive applications has been demonstrated [8,9,10].

There are industrial standards specifying the requirements for breath analyzers intended for evidential, professional and consumer applications, including alcohol interlocks [11,12,13,14]. In a previous publication [10], it was demonstrated that the technology based on infrared spectroscopy is capable of fulfilling these requirements.

In rapid screening applications there are additional requirements on throughput, ease of use, and routines for dealing with occasional “red flags”, *i.e.*, BrAC values above a preset threshold. The required throughput may vary somewhat from one scenario to another, but as a general rule the overall time allocated for each breath test should not exceed 10 seconds. This rule will, in practice, prohibit the use of disposable mouthpieces.

Physiological and behavioral influences are known to outnumber other error sources [6,9,10]. This paper actually represents an attempt at coming to grips with such influences by introducing algorithms which could potentially reduce their effects. The approach is based on the observation that significant variability contributions are attributed to the dilution of the breath sample, and inter-individual variability [10].

This paper is focusing on algorithm development and addressing these variations and thereby improving rapid breath alcohol screening with respect to accuracy. An outline of methods and technology being used in the development work is provided, followed by results on various algorithm implementations using data from a previous human study [10]. Concluding remarks are thereafter provided.

## 2. Methods and Technology

The requirements for breath alcohol screening as described in the previous section excludes the use of disposable mouthpieces which are normally associated with breath alcohol determination. Measurement is performed at a sensor location in the proximity of the subject’s mouth and nose, where a relatively high concentration of exhaled breath occurs either due to forced or spontaneous breathing.

In the system used in this study, the dilution of exhaled air with ambient air is estimated by simultaneous measurement of carbon dioxide at basically the same sensing location as the alcohol detection. The sensor system includes an air inlet defining the sampling point at which air is continuously being withdrawn, and fed to a measuring cell including optical and sensing elements for real-time infrared transmission measurement for the selective detection of CO${}_{2}$ and EtOH, respectively. The sensor signals having a sampling rate of 5 samples per second are digitized into a standard, calibrated format corresponding to local gas concentrations.

**Figure 1:**
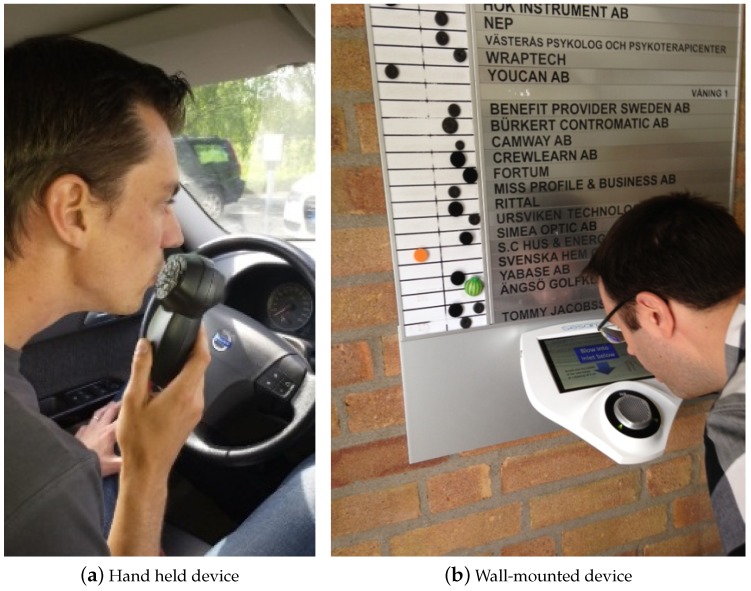
(**a**) Hand held prototype device for automotive breath alcohol screening; (**b**) Wall mounted device for passage control and workplace breath alcohol screening.

The complete breath analyzer is assembled within a housing allowing handheld or wall-mounted use as shown in Figure 1. The wall-mounted analyzer includes a touch-screen for user interaction. More detailed descriptions of the sensor design have been published elsewhere [9,10].

The occurrence of a CO${}_{2}$ peak is used as an indicator of a breath above the background level. If a corresponding peak of ethyl alcohol (EtOH) is detected at basically the same point in time, it is possible to estimate BrAC using the following equation, Equation (1).

(1) $$BrAC=EtOH_{meas}*DF=EtOH_{meas}*\frac{CO_{2et}-CO_{2background}}{CO_{2meas}-CO_{2background}}$$

The subscript “meas” denotes the measured peak values, and “CO${}_{2et}$” the end tidal CO${}_{2}$ concentration, which is believed to approach the alveolar concentration, typically 4.8 ± 0.5 vol% [7,15]. DF is the dilution factor, ranging from one in highly concentrated air close to the mouth of the subject, to large numbers at large distances. The background CO${}_{2}$ concentration is typically less than 0.1 vol% [16]. The influence of CO${}_{2}$ background variations over that range on the dilution factor is then less than 3.2% according to Equation (1). In the present investigation CO${}_{2background}$ was set to zero. The standard measurement unit for BrAC is mg/L, which relates to blood alcohol concentration (BAC, %) by the approximate relation 1 mg/L BrAC = 0.2%BAC [13]. The US legal limit of 0.08%BAC thus corresponds to a BrAC value of 0.4 mg/L.

An error in the estimation of CO${}_{2et}$ will evidently have a direct impact on the BrAC estimation according to Equation (1). As described in previous publications [6,10], the present implementation involves a number of sources of variability of physiological origin, such as anatomy and efficiency of gas exchange within the respiratory tract. Such variations will inevitably lead to inter-individual variability of the estimated end-tidal concentration. In addition, there will be circumstantial variability depending on the subject’s breathing pattern at or immediately before the actual breath sample.

In an attempt to reduce the impact of these variations, the algorithms for BrAC estimation investigated in this paper include the following characteristics: (i) Signal averaging over several signal samples for stochastic noise suppression in combination with Equation (1); (ii) a weighted average between Equation (1) and ETOH${}_{meas}$, to address inter-individual variation; and (iii) personalization, taking individual variations of CO${}_{2et}$ into account. In the present study, the noise suppressing algorithm (i) is used as “baseline” from which possible improvement due to weighting (ii) and personalization (iii) is evaluated. It should be noted that the same averaging technique, which consists of a moving average filter, is used in all three algorithms.

The following equation, Equation (2), was used to calculate the weighted average (ii).

(2) $$BrAC=\frac{1}{DF}*EtOH_{meas}+\left(1-\frac{1}{DF}\right)*BrAC$$

In Equation (2) the impact of the tracer gas is lower for less diluted samples. The third calculation method evaluated in this paper, personalization (iii), utilizes individual end tidal CO${}_{2}$ concentration. Compared to Equation (1) instead of using a generic factor for CO${}_{2et}$ for all individuals, the end tidal value for each subject was measured and used in the analysis. The altered equation, Equation (3), follows.

(3) $$BrAC_{Ind}=EtOH_{meas}*DF=EtOH_{meas}*\frac{CO_{2etInd}-CO_{2background}}{CO_{2meas}-CO_{2background}}$$

The data used for evaluation of the alternative algorithms were taken from a human study involving 30 test subjects and a total of 658 breath tests. In the human study, each breath test performed with the prototype devices was directly followed by a breath test with an evidential breath analyzer (Evidenzer, Nanopuls AB, Uppsala, Sweden) providing a reference BrAC value. More detailed descriptions of the human study have been published elsewhere [9,10]. In the study, each subject was dosed with alcohol based on the subjects body weight. 0.6 grams of ethyl alcohol per kilogram of body weight were given to male subjects and 0.55 grams of ethyl alcohol per kilogram of body weight were given to female subjects. The alcohol was consumed in less than 15 min. Measurements were performed in sets, given in Table 1, during the alcohol elimination phase. One measurement set was repeated every 20 min. The study was approved by the Swedish Ethical Review Board in Uppsala (Dnr 2013/089).

The present investigation is focused on situations with moderate dilution, corresponding to a sensor location at less than 5 cm distance from the mouth/nose region of the test subject. This corresponds to a dilution factor DF < 5 according to Equation (1). Hence, recordings from prototype tests performed at 3 cm and 15 cm with measured DF < 5 was included in the analysis and compared to reference instrument recordings collected during the same measurement set.

## 3. Results

In this section, graphs of recorded breath test signals are provided to visualize the influence of dilution on the signal quality. The results of applying the algorithms (i), (ii), and (iii) to signals recorded during the human study are then presented.

**Figure 2:**
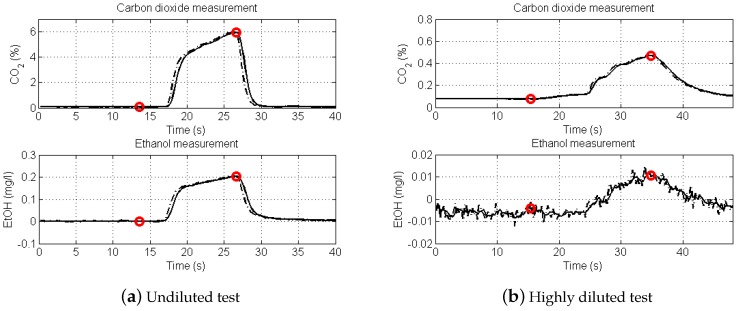
Graphs of signals from two breath tests of intoxicated subjects. (**a**): Undiluted breath test. (**b**): Diluted breath test. Both tests were performed with an intoxication level close to 0.20 mg/L.

Figure 2 shows recorded breath test signals from two intoxicated subjects. The left graphs are simultaneous recordings of CO${}_{2}$ (upper tracing) and EtOH (lower tracing) of an undiluted breath test (DF = 1). Note the similarity of the CO${}_{2}$ and EtOH waveforms.

The recording at right shows the corresponding signals at high dilution, DF = 14. Using Equation (1) to determine BrAC provides 0.20 and 0.21 mg/L, respectively, for the undiluted and highly diluted estimations. The red circles indicate signal sampling points used in the BrAC calculation. The signal to noise ratio of the highly diluted EtOH signal is <10.

Signal recordings from the human study were used for comparison of the three algorithms (i), (ii), (iii) for BrAC estimation in the DF range 1 to 5. The results are summarized in Table 1 and Figure 2.

**Figure 3:**
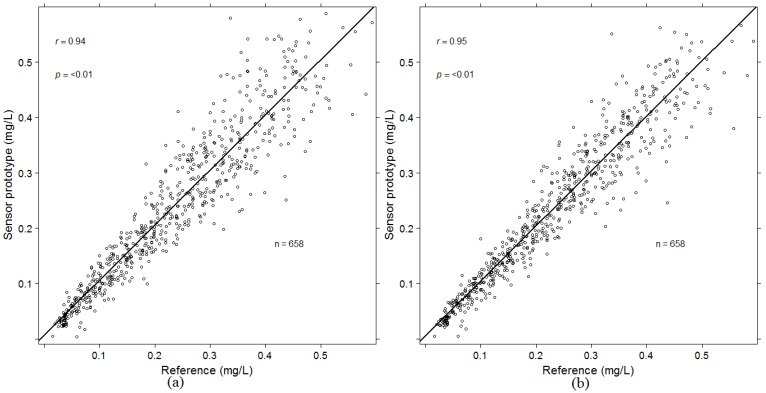
Graphs of breath alcohol concentration (BrAC) determination *versus* reference values using the averaging algorithm (i) (**a**), and all (i), (ii), (iii) combined (**b**). The variation around the identity line is visibly reduced in the right graph.

Simply by using Equation (1) there is a random estimation error of the averaging algorithm (i) largely proportional to the alcohol concentration as illustrated in the left graph of Figure 3. The one standard deviation (1*σ*) magnitude of the error is approximately 25%.

A quantitative comparison of the random estimation errors of the algorithms (i), (ii), and (iii) is provided in Table 1. The random error is significantly reduced by both algorithms (ii) and (iii) from 25% to 19% and 18% respectively. If all algorithms are combined, the random error is reduced even further, to 15%. The error reduction is visualized by comparing the left and right graphs of Figure 3. The right hand side corresponds to the combined algorithm. The error reduction is also presented in Table 2.

## 4. Discussion

At high levels of dilution, signal to noise ratio becomes a significant error contribution as indicated in the right graph of Figure 2. Therefore, an upper limit of DF = 5 was set in the algorithm evaluation.

The observed variability of BrAC determination in the screening mode (Figure 3) at moderate dilution is mainly attributed to the variability of the estimated CO${}_{2et}$ [6,10]. The approach of the weighted algorithm (ii) is to decrease the influence of this variability at moderate dilution, whereas the personalized algorithm (iii) aims at minimizing the influence of individual variability.

A clear demonstration that significant improvement of accuracy in the screening mode can be obtained by algorithms (ii) and (iii) taken alone or in combination (Table 2). The 1*σ* variability was reduced from 25% to 15 %.

The results enable more accurate determination of breath alcohol in moderately diluted samples and thus truly positive or truly negative classifications can be made more reliably. In any application where a directed breath can be applied, e.g., everyday use in passage controls or prevention of alcohol impaired driving, such techniques as described herein are favourable. Other areas, such as evidential breath testing, may still require even higher accuracy and may very well require a breath test using a mouthpiece. A methodology using a two step procedure has previously been published on the subject [10]. In the proposed methodology breath alcohol screening techniques can be used up until a measurement is close to the legal limit, only then a high accuracy technique is required.

Less obtrusive solutions will gain more acceptance from a general public, especially in automotive applications for driver monitoring. Further development regarding technology and algorithms will be needed to reliably measure and evaluate breath alcohol passively.

## 5. Conclusions

The random error in contact free breath alcohol determination can be reduced by up to 40% by utilizing algorithms presented in this paper.

## Figures and Tables

**Table 1:** Measurement set used during the human subject study.

Test Order	Instrument	Execution
1	Reference instrument	Mouthpiece
2	Prototype	Mouthpiece
3	Prototype	3 cm distance
4	Prototype	15 cm distance

**Table 2:** Comparison of estimation errors of the three algorithms. Weighting and personalization alone decrease the random estimation by 24% respectively 28%. They can also be used in combination and thereby further reduce the random estimation error by up to 40%.

Algorithm	Random Estimation Errors (1*σ*, %)	Random Errors Relative to (i)
Averaging (i)	25	1
Weighting (ii)	19	0.76
Personalization (iii)	18	0.72
All combined	15	0.60
